# PCR-Based Molecular Authentication Method for Sources of Agrimoniae Herba via Comparative Analyses of Complete Chloroplast Genomes

**DOI:** 10.3390/ijms262211189

**Published:** 2025-11-19

**Authors:** Woojong Jang, Sae Hyun Lee, Wook Jin Kim, Sungyu Yang, Byeong Cheol Moon

**Affiliations:** 1 Herbal Medicine Resources Research Center, Korea Institute of Oriental Medicine, Naju 58245, Republic of Korea; wjjang@kiom.re.kr (W.J.); ukgene@kiom.re.kr (W.J.K.); sgyang81@kiom.re.kr (S.Y.); 2 Texas A&M AgriLife Research and Extension Center, Dallas, TX 75252, USA; saehyun.lee@ag.tamu.edu

**Keywords:** Agrimoniae Herba, *Agrimonia*, chloroplast genome, molecular authentication marker, species identification

## Abstract

Accurate species identification is essential for the quality control and standardization of herbal medicines. *Agrimonia* species, the authentic sources of Agrimoniae Herba, have long been used in traditional medicine, yet limited genomic resources have hindered the establishment of reliable molecular approaches for accurate species discrimination within this genus. Here, we report the newly assembled complete chloroplast genomes (155,156–155,302 bp) of four *Agrimonia* species, which exhibit the typical quadripartite structure and contain 112 unique genes. Comparative analysis revealed 684 variable sites, including 497 single nucleotide polymorphisms (SNPs) and 187 insertions/deletions (InDels), predominantly located in the single-copy regions. Based on these species-specific variations, we developed nine PCR-based molecular markers that distinguished the four species. The markers were validated using herbarium specimens and commercial herbal products, demonstrating reproducibility and practical applicability. Phylogenetic analysis supported the monophyly of the genus *Agrimonia* and resolved each species into distinct clusters within the subtribe Agrimoniinae. These results showed that chloroplast genome sequences of the genus *Agrimonia* can serve as effective super DNA barcodes for species identification. Our study provides fundamental genomic resources for *Agrimonia* and reliable molecular tools for species authentication, providing a basis for ensuring the authenticity and safety of Agrimoniae Herba.

## 1. Introduction

The genus *Agrimonia* (Rosaceae), including 12–15 species, is widely distributed across the temperate regions of the Northern Hemisphere [1]. These perennial herbaceous flowering plants have been used as traditional medicinal herbs for centuries [2,3]. In East Asia, where traditional medicine is well established, species in *Agrimonia* are officially designated as herbal medicines and remain widely utilized. Agrimoniae Herba contains over 100 secondary metabolites with anti-inflammatory, antioxidant, and anticancer effects [4], and its pharmacological potential is an active area of research. Owing to its remarkable pharmacological efficacy, research has focused on its therapeutic effects and related bioactive compounds; however, genomic studies remain relatively limited.

In Asian countries, including Korea and China, the use of officially designated authentic species or taxa is strictly regulated for medical herbal medicines to ensure therapeutic equivalence and consistency in clinical efficacy. Ensuring the authenticity of herbal materials is particularly important, as the use of incorrect species can cause significant health and social issues. However, most herbal materials are processed or sliced prior to distribution, making it difficult to verify whether the correct species have been used. Therefore, accurate species identification is a critical prerequisite for the quality control and standardization of herbal medicines. In such cases, molecular markers that enable rapid and accurate species identification through simple polymerase chain reaction (PCR) amplification are highly valuable for preventing the distribution of adulterated or substituted herbal materials. Traditionally, species identification has been performed using DNA barcoding based on the internal transcribed spacer (ITS) region or partial chloroplast genome sequences, such as *matK* or *rbcL* [5]. These methods rely on limited sequence information and provide low resolution. Recent advances in sequencing and related analysis technologies now allow the treatment of large-scale sequence data, enabling comparisons of more comprehensive genomic information.

In plants, the chloroplast is a unique organelle that plays essential roles in photosynthesis and is indispensable for survival [6]. It possesses a small, relatively independent genome, which is highly conserved and stably inherited across generations [7]. The low level of sequence variation within species is valuable for species identification [8], phylogenetic and evolutionary analyses [9], genetic diversity assessment [10], and molecular marker development [11]. Complete chloroplast genomes can be readily assembled, and reliable molecular markers capable of distinguishing species across multiple regions can be developed based on genome-wide comparisons. However, to date, no molecular markers derived from chloroplast genome data have been developed for the discrimination of *Agrimonia* species.

In this study, we aimed to develop molecular marker set using chloroplast genome information of *Agrimonia* species for accurate species identification, thereby contributing to the quality control and standardization of Agrimoniae Herba. We collected four *Agrimonia* species (*A. coreana*, *A. gorovoii*, *A. nipponica*, and *A. pilosa*) distributed in Korea and assembled their complete chloroplast genome sequences. Comparative analyses were carried out to identify species-specific variants, from which molecular markers for species authentication were developed. In addition, phylogenetic analyses were conducted to clarify the evolutionary relationships and assess the potential of chloroplast genomes as super DNA barcodes for species discrimination within the genus *Agrimonia* and closely related taxa. These results provide fundamental genomic resources for an underexplored genus as well as species-specific molecular markers for ensuring the reliability of the herbal medicine distribution system.

## 2. Results

### 2.1. Complete Chloroplast Genomes of Agrimonia Species

**Figure 1:**
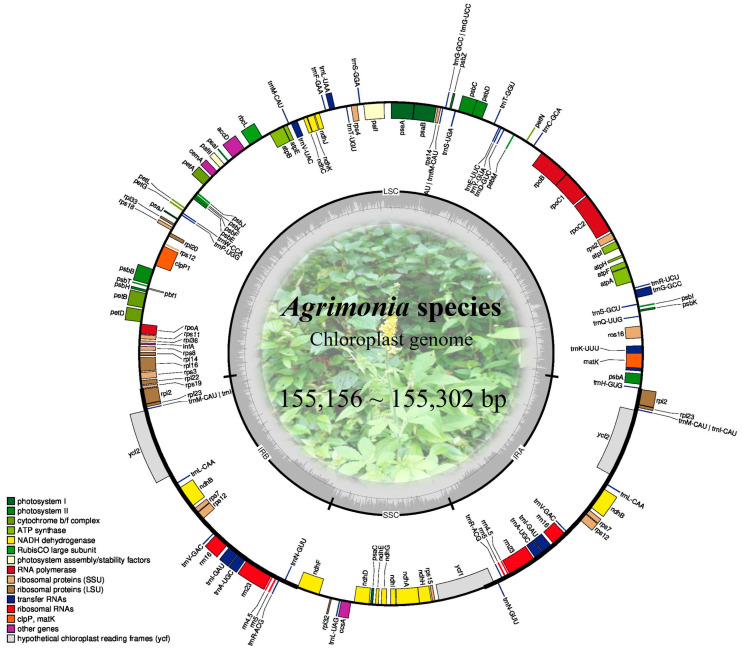
Circular gene map of chloroplast genomes for *Agrimonia* species. Genes inside and outside of the circle are transcribed clockwise and counterclockwise, respectively. The dark gray portion of the inner circle represents the GC content.

Whole genome sequencing of four *Agrimonia* species distributed in Korea generated 7.9–15.3 million sequencing reads with an average length of 300 bp (Table S1). The filtered paired-end reads provided sufficient coverage to assemble complete chloroplast genomes ranging from 155,156 to 155,302 bp in length (Table 1). Among the filtered data, 0.74–1.43% of the reads were mapped to the chloroplast genomes, resulting in a sequencing depth of 70× to 185× (Table S1). The assembled genomes exhibited the typical quadripartite structure, consisting of two inverted repeats (IRa and IRb, 25,954–25,965 bp) separating the large single-copy (LSC, 84,489–84,650 bp) and small single-copy (SSC, 18,723–18,744 bp) regions (Table 1). The genomes contained 112 unique genes, including 79 protein-coding genes, 29 tRNA genes, and 4 rRNA genes, with an overall GC content of 36.9% (Table 1, Figure 1).

### 2.2. Sequence Variation Among Agrimonia Species

**Figure 2:**
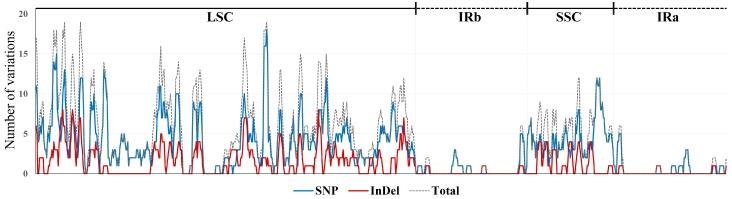
Variable positions in the chloroplast genomes of *Agrimonia* species determined using a sliding-window approach with a window size of 1000 bp and step size of 200 bp.

Comparative analyses of the four *Agrimonia* chloroplast genomes revealed 684 variable sites, including 497 single nucleotide polymorphisms (SNPs), which represent single-base substitutions, and 187 insertions/deletions (InDels), which correspond to sequence insertion or losses (Table 2). Pairwise comparisons revealed the smallest number of SNPs (239) between *A. gorovoii* and *A. pilosa*, whereas the largest number (302) was identified between *A. coreana* and *A. nipponica*. For InDels, the fewest variants (102 sites, totaling 483 bp) were observed between *A. nipponica* and *A. pilosa*, while the greatest number of InDel sites (119) occurred between *A. gorovoii* and *A. nipponica*. However, the longest total length of InDel variation (840 bp) was identified between *A. coreana* and *A. gorovoii*. The total variants (SNPs + InDels) were smallest (346 sites) between *A. coreana* and *A. pilosa* and largest (419 sites) between *A. coreana* and *A. nipponica*. Species-specific sequences, which are particularly valuable for developing molecular markers for species authentication, were least frequent (143 sites) in *A. pilosa* and most abundant (211 sites) in *A. nipponica* (Table 2). Most of the polymorphic sites were located within the LSC and SSC regions, whereas relatively few were detected in the IR regions (Figure 2).

### 2.3. Development and Application of Species-Specific Markers

**Figure 3:**
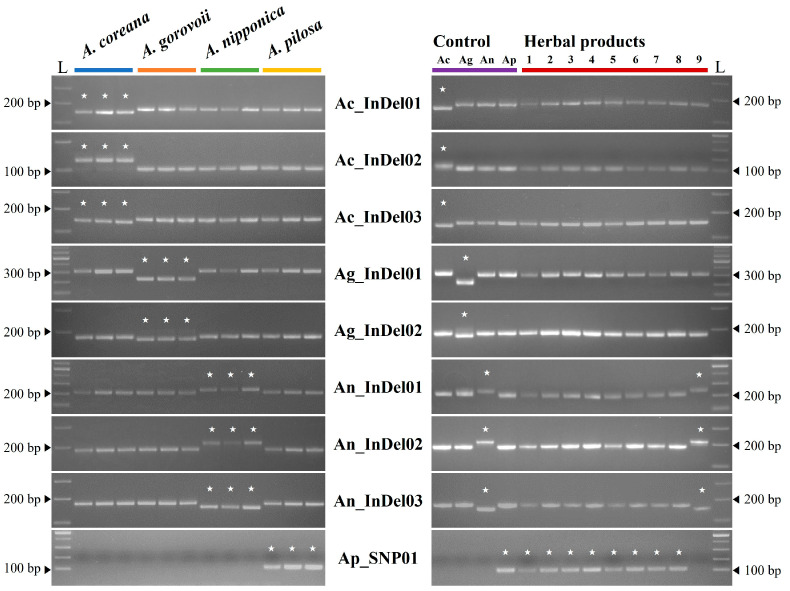
Validation and application of molecular markers for genetic authentication of four *Agrimonia* species. The left-hand panel shows the results for the application of the developed molecular markers to herbarium specimen, and the right shows the results for herbal products. The list of herbal products (1–9) can be found in Table S2. * Ac: *A. coreana*, Ag: *A. gorovoii*, An: *A. nipponica*, Ap: *A. pilosa*, L: ladder.

Based on the species-specific sequences identified from genome-wide comparisons, primer pairs were designed to target long InDel regions, which are generally expected to exhibit little intraspecific variation. For *A. pilosa*, where no suitable InDel regions were available, primer pairs were developed from non-synonymous SNP sites. When tested on several specimens for each species, nine primer sets, excluding a few that showed intraspecific variation, produced the expected polymorphic patterns (Table 3, Figure 3). The co-dominant InDel markers (Ac_InDel, Ag_InDel, and An_InDel) amplified products across all four species; however, the target species consistently yielded fragments of distinct sizes, highlighting interspecific differences. The SNP marker AP_SNP01, developed as a dominant marker, produced amplicons only in *A. pilosa*. All markers amplified fragments of the predicted sizes from the respective chloroplast genome sequences and clearly distinguished the target species among *Agrimonia* plants. In addition, the developed molecular markers were applied to commercial herbal products randomly obtained from the market (Figure 3). Samples 1–8 were identified as *A. pilosa*, while sample 9 was identified as *A. nipponica*. Each marker detected only a single species for each sample, with no evidence of mixed herbal materials. These findings indicate that most Agrimoniae Herba currently distributed in the market are primarily produced from *A. pilosa*.

### 2.4. Phylogenetic Relationships Among Agrimonia and Related Taxa

**Figure 4:**
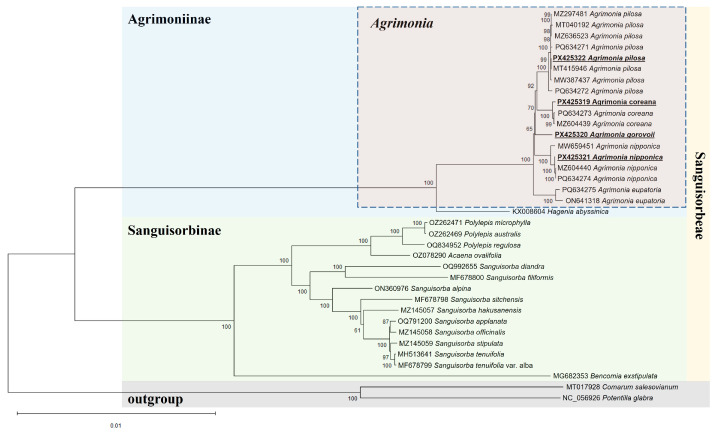
Phylogenetic tree of *Agrimonia* and related species based on the whole sequences of chloroplast genomes. Numbers at nodes indicate bootstrap support based on 1000 replicates. Scale bar represents 0.01 substitutions per site. Underlined bold texts indicate the chloroplast genomes assembled in this study.

Phylogenetic analysis was performed to examine the basic evolutionary relationships of the *Agrimonia* genus and to evaluate the utility of chloroplast genome sequences for species identification. A phylogenetic tree was reconstructed using 34 chloroplast genome sequences from species of the tribe Sanguisorbeae and two outgroup sequences (Figure 4). The tree included 15 sequences for six species in the subtribe Agrimoniinae and 15 sequences for the subtribe Sanguisorbinae, together with the four chloroplast genomes of *Agrimonia* obtained in this study. All species were clearly divided into two major clades corresponding to Agrimoniinae and Sanguisorbinae in the phylogenetic tree. Species in the genus *Agrimonia* formed a monophyletic group, and each species formed a distinct cluster within the Agrimoniinae clade. The four sequences for the *Agrimonia* species reported in this study were placed appropriately within their respective clusters. These results suggested that chloroplast genome sequences of the genus *Agrimonia* can be effectively utilized not only to elucidate their evolutionary relationships but also as super DNA barcodes for accurate species identification.

## 3. Discussion

We sequenced the complete chloroplast genomes of *A. coreana*, *A*, *gorovoii*, *A. nipponica*, and *A. pilosa*, providing comprehensive genomic resources for *Agrimonia*. This study addresses the limited fundamental genomic information for the genus, with only sporadic studies of *A. pilosa* and a lack of data for most other species [12,13]. The four chloroplast genomes exhibited the typical quadripartite structure found in most angiosperms and showed highly conserved sizes. Comparative analyses revealed 684 variable sites, concentrated in the LSC and SSC regions, with highly conserved IR regions, consistent with observations in other angiosperm genomes [7,14]. The high conservation of chloroplast genomes is generally attributed to their essential roles in photosynthesis and other fundamental metabolic processes, which imposes strong selective constraints on sequence variation [15]. In particular, levels of conservation are higher in the IR regions than in the LSC and SSC regions, likely due to their involvement in stabilizing the chloroplast genome structure through homologous recombination and copy correction mechanisms [16].

The variations identified in the chloroplast genomes also provided valuable insights into the fundamental genomic characteristics of each species. We detected a pseudogene in both *A. nipponica* and *A. pilosa*, corresponding to *infA* and *rps16*, respectively. These genes were nonfunctional due to the emergence of early stop codons caused by sequence insertions and substitutions. In many angiosperms, pseudogenes are functionally compensated by the transfer of homologous genes to the nuclear genome, where they continue to perform essential functions [17]. Pseudogenization events are frequently reported in land plants [18,19,20]. Within the genus *Agrimonia*, they may be regarded as species-specific genomic traits.

Ensuring the equivalence of efficacy and consistency of therapeutic effects in herbal medicines requires the use of authentic plant species listed in the pharmacopeia. Species-specific variations in chloroplast genome sequences provide key information for species authentication, and some of these variants can be applied to the development of molecular markers. PCR-based molecular markers that enable rapid and accurate species identification are valuable tools for ensuring the authenticity and safety of herbal materials by preventing adulteration or substitution. In this study, nine markers that successfully distinguished the four *Agrimonia* species were developed, representing the first report of chloroplast genome–based markers for *Agrimonia* species identification. Their polymorphisms were validated using *Agrimonia* specimens collected from various natural habitats, and their reproducibility and applicability were further demonstrated through tests on commercial herbal products. The absence of cross-amplification among multiple random samples confirmed that the developed markers reliably discriminated the four species within the genus *Agrimonia*.

Herbal medicines are often subject to mislabeling because authentic species and adulterants can be morphologically similar, and their processed forms, such as sliced or ground materials, make accurate identification particularly challenging. In addition, pharmacopeial regulations differ among countries, leading to inconsistencies in the scope of acceptable herbal resources. For example, while only *A. pilosa* is permitted as authentic Agrimoniae Herba in China and Taiwan, Korea allows the use of *A. pilosa* together with closely related species in the genus. Such discrepancies, combined with the difficulty of morphological identification, highlight the need for a reliable system to ensure the authenticity and safety of herbal medicines in the marketplace. In this context, species-specific molecular markers represent an effective alternative. The PCR-based marker set developed in this study demonstrated clear discrimination of the four *Agrimonia* species, both in reference specimens and in commercial products, thereby underscoring its practical utility. These markers may also provide particularly valuable information in countries such as China and Taiwan, where the definition of authentic Agrimoniae Herba is restricted to a narrow species range. However, it should be noted that the present analyses and marker validation were limited to four *Agrimonia* species collected from Korea. When applied to other closely related taxa, there remains a potential risk of cross-amplification or reduced marker specificity. To overcome these limitations, further comparative analyses involving a broader range of species and geographically diverse samples are required.

A phylogenetic analysis based on complete chloroplast genome sequences clearly supported the division of the subtribes Agrimoniinae and Sanguisorbinae within the tribe Sanguisorbeae as well as the monophyly of the genus *Agrimonia*. These results are consistent with those of previous phylogenetic studies using nuclear and partial chloroplast markers [21]; however, the use of whole chloroplast genomes in the present study provided greater resolution and stronger support for interspecific relationships. Furthermore, the arrangement of the four newly reported *Agrimonia* chloroplast genomes within their corresponding species clusters in the phylogenetic tree supported the use of complete chloroplast genomes as effective super DNA barcodes for accurate species identification. This indicates that chloroplast genome sequences alone provide sufficient resolution to discriminate among species [22]. The overall phylogenetic framework presented here provides valuable insights into the evolutionary relationships within *Agrimonia* and offers a genomic foundation for future taxonomic and phylogeographic investigations.

## 4. Materials and Methods

### 4.1. Plant Materials and DNA Extraction

Four species in the genus *Agrimonia* (*A. coreana*, *A. gorovoii*, *A. nipponica*, and *A. pilosa*) were collected from natural habitats in Korea (Table S2). All collected plant materials were taxonomically identified and verified by a taxonomist, Dr. Sungyu Yang, before further processing. The plants were made into voucher specimens and were registered with the Korean Herbarium of Standard Herbal Resources (Index Herbariorum Code KIOM) at the Korea Institute of Oriental Medicine (KIOM, https://oasis.kiom.re.kr/herblib/ (7 October 2025)). A representative individual for each species was selected for chloroplast genome sequencing (Table S2). Fresh leaves of each plant were ground in liquid nitrogen using a mortar and pestle, and genomic DNA was extracted using the DNeasy^®^ Plant Mini Kit (Qiagen, Hilden, Germany) in accordance with the manufacturer’s instructions. DNA quality and quantity were measured using an ND-1000 UV/Vis spectrophotometer (NanoDrop, Wilmington, DE, USA).

### 4.2. Genome Sequencing and Assembly

Sequencing libraries were prepared using the TruSeq Nano DNA Kit (Illumina Inc., San Diego, CA, USA) in accordance with the manufacturer’s instructions. Whole genome sequencing was performed based on a multiplexing method using the MiSeq platform (Illumina Inc.) to generate 300 bp paired-end reads. The reads were sorted according to index sequences for each species. Raw data were trimmed using trimmomatic v0.39 [23] with the following settings: Phred score: 33, LEADING: 30, TRAILING: 30, SLIDINGWINDOW: 4:15, and MINLEN: 80. Chloroplast genome assembly was performed using NOVOPlasty v4.3.1 [24] and GetOrganelle v1.7.7.1 [25] based on a reference sequence (*A. pilosa*, GenBank accession no. NC050051) with default parameters. The assembled sequences were validated through raw read mapping using bwa v0.7.17 [26] and samtools v1.9 [27], particularly to confirm the accuracy of the boundary regions in the chloroplast genomes. The chloroplast genomes were annotated using GeSeq v2.03 [28] and manual curation was performed using Artemis v18.2.0 [29] based on the reference sequences. The composition of the chloroplast genomes was visualized using OGDRAW v1.3.1 [30]. All raw sequencing data, chloroplast genome sequences, and annotation data were deposited in a public database at the National Center for Biotechnology Information (NCBI, https://www.ncbi.nlm.nih.gov/ (7 October 2025)).

### 4.3. Comparative Analysis

A multiple sequence alignment of the completed chloroplast genomes was generated using MAFFT v7.526 [31] with default parameters. After manual curation of the detailed alignments, SNPs and InDels among the species were identified from the filtered data using the conditional (IF) function in Microsoft Excel 2021 (Redmond, WA, USA). The sliding-window analysis was applied to examine the distribution pattern of the variants across the chloroplast genome, with a window size of 1000 bp and step size of 200 bp. A phylogenetic analysis was conducted based on the complete chloroplast genome sequences of the four *Agrimonia* species, together with sequences for 14 *Agrimonia* species and 16 representative species belonging to the tribe Sanguisorbeae registered in NCBI. Sequences from the genus *Comarum* and *Potentilla* were included as outgroups. In total, 36 chloroplast genome sequences were aligned using MAFFT v7.526 [31], and a phylogenetic tree was reconstructed using the maximum-likelihood method and the Kimura two-parameter model [32] with 1000 bootstrap replicates in MEGA12 [33].

### 4.4. Marker Development and Application

Species-specific PCR primers were designed for non-synonymous SNPs and InDels, excluding simple sequence repeats, and focusing on InDels showing interspecific length differences of at least 7 bp. The designed primer pairs were validated using Primer-BLAST, https://www.ncbi.nlm.nih.gov/tools/primer-blast/ (7 October 2025), to check for potential primer–dimer formation and nonspecific amplification. The validated primer pairs were applied to 12 specimens of *Agrimonia* deposited in the herbarium at KIOM and to nine commercial herbal medicines sold as Agrimoniae Herba (Table S2). PCR amplification was carried out in a 40 μL volume containing 1X Taq PCR Smart mix (Solgent, Daejeon, Korea), 10 ng of genomic DNA, and 10 pmole of each primer. The following thermal cycle conditions were used: 10 min at 95 °C for denaturation; 35 cycles of 20 s at 95 °C, 20 s at the optimal annealing temperature, and 20 s at 72 °C; and 7 min at 72 °C for the final extension. The optimal annealing temperature for each primer pair was determined through gradient PCR at 56–65 °C. Marker polymorphism was evaluated by electrophoresis for 1–2 h using 1.5% and 3% agarose gels.

## 5. Conclusions

In this study, we obtained complete chloroplast genome sequences for four *Agrimonia* species. Through comprehensive comparative analyses, interspecific variation across the chloroplast genomes was identified, and species-specific polymorphisms were applied to develop PCR-based molecular markers capable of distinguishing the four species. The developed markers were validated using *Agrimonia* specimens for each species collected from diverse natural habitats as well as commercial herbal products, thereby demonstrating their reproducibility and practical utility in species identification. Although the present results may involve potential risks associated with the limited geographic diversity of the sampled materials and the lack of evaluation against additional closely related species, the developed markers successfully distinguished the four *Agrimonia* species used as authentic sources of Agrimoniae Herba in Korea, confirming their robustness and applicability. They will contribute substantially to reducing the risk of adulteration and enhancing the reliability of herbal medicines, for which the use of authentic species is critical. Furthermore, the newly reported fundamental genomic resources for this underexplored genus will provide valuable insights into understanding its genetic characteristics.

## Figures and Tables

**Table 1:** Structure and features of chloroplast genomes for *Agrimonia* species assembled in this study.

Species Name	Length (bp)				Number of Unique Genes (PCG/tRNA/rRNA)	GC Content (%)	GenBank Accession No.
	LSC	SSC	IR	Entire			
*A. coreana*	84,650	18,744	25,954	155,302	112 (79/29/4)	36.88	PX425319
*A. gorovoii*	84,560	18,741	25,955	155,211		36.90	PX425320
*A. nipponica*	84,514	18,723	25,962	155,161		36.90	PX425321
*A. pilosa*	84,489	18,737	25,965	155,156		36.91	PX425322

IR: inverted repeat; LSC: long single-copy; PCG: protein-coding gene; SSC: short single-copy.

**Table 2:** Summary of the variants identified in the chloroplast genomes of *Agrimonia* species.

		SNPs				Total: 497			
		*A. coreana*		*A. gorovoii*		*A. nipponica*		*A. pilosa*	
**InDels**	***A. coreana***		118	267		302		241	
		61 (354 bp)							
	***A. gorovoii***	113 (840 bp)			109	283		239	
				62 (423 bp)					
**Total** **187** **(1333 bp)**	***A. nipponica***	117 (702 bp)		119 (726 bp)			148	278	
						63 (219 bp)			
	***A. pilosa***	105 (581 bp)		108 (691 bp)		102 (483 bp)			96
								47 (144 bp)	

InDel values indicate the number of variant sites, with the total length (bp) in parentheses. The numbers in gray cells indicate the number of species-specific variations.

**Table 3:** Details for the molecular markers developed to authenticate *Agrimonia* species.

Marker Name	Target Species	Target Region			Primer Sequence (5′ -> 3′)		Annealing Temperature (°C)	Product Size (bp)	
		Locus		Position *					
Ac_InDel01	*A. coreana*	LSC	*atpB*–*rbcL*	54,969/54,970	F	GAACCCGATTCCATTGTTTACTT	56	T	155
					R	GAAGGTGTTGTCTATAATGATAGGC		O	172
Ac_InDel02	*A. coreana*	LSC	*trnP*(UGG) –*psaJ*	66,773–66,800	F	CGCTACATCCCTTTCAATTTG	58	T	130
					R	AGATATTTTATTTTGGTATACCAATGGAAG		O	102
Ac_InDel03	*A. coreana*	IR	*trnR*(ACG) –*trnN*(GUU)	108,808/108,809	F	CTATGGAATTTTGGCGTGGGT	56	T	144
				131,131/131,312	R	CGGGGCGTAAAAGTAAAACAT		O	155
Ag_InDel01	*A. gorovoii*	LSC	*psbM* –*trnD*(GUC)	30,206/30,207	F	ATGATTGAACCGCCCCTACA	56	T	226
					R	AGAATACTTCCAGGAATCGCC		O	306–313
Ag_InDel02	*A. gorovoii*	IR	*ycf2*	87,607/87,608	F	TGAATTCGGGACAGCTATTCG	60	T	173
				152,163/152,164	R	AGCCCACTTGTTTCTCGAGA		O	182
An_InDel01	*A. nipponica*	LSC	*trnS*(GCU) –*trnG*(GCC)	8731–8760	F	CTGGTCAGTACTTAGCCGGG	56	T	246
					R	TGTATTGTGCTAAGAAACGCGA		O	215–219
An_InDel02	*A. nipponica*	LSC	*trnV*(UAC) –*trnM*(CAU)	52,387/52,388	F	ATTGGCGCGCGTGTAAAC	56	T	152
					R	ACTTATAAGCAATACCGATCAAACAGA		O	170
An_InDel03	*A. nipponica*	LSC	*infA*	79,995–80,005 and 80,059–80,075	F	GTATCCCTCCGAAAGAATGTTGAA	56	T	215
					R	GGTTTTGCTTCGGAAAAGGTTC		O	187
Ap_SNP01	*A. pilosa*	LSC	*rps16*	5051	F	GTTTGTTGATTAAGGCGAAGTGA	59	T	105
					R	TGCTATTCTATACTTCCTTGAAAAGG		O	-

* Position in the chloroplast genome corresponding to the target species for each marker. Underlined bold text in Ap_SNP01 primer sequence indicates modified nucleotide from the template to enhance species-specificity. F: forward; O: others; R: reverse; T: target.

## Data Availability

The sequencing reads have been deposited in the NCBI database under BioProject accession no. PRJNA1332120; the link is https://www.ncbi.nlm.nih.gov/bioproject/PRJNA1332120 (7 October 2025, the SRA accession nos. for *A. coreana*, *A. gorovoii*, *A nipponica*, and *A. pilosa* are SRR35570240, SRR35570237, SRR35570238, and SRR35570239, respectively). Chloroplast genome sequences and annotation data for each species produced from this article can be found in the GenBank data libraries under the accession numbers PX425319, PX425320, PX425321, and PX425322, respectively.

## Supplementary Materials

The following supporting information can be downloaded at: https://www.mdpi.com/article/10.3390/ijms262211189/s1.
